# Boundary issues in regulation and evolution of wood formation

**DOI:** 10.1186/1753-6561-5-S7-O36

**Published:** 2011-09-13

**Authors:** Yordaan Yordanov, Victor Busov

**Affiliations:** 1 Forest Resources and Environmental Science, Biotechnology Research Center, Michigan Technological University, USA

## 

Wood has played an indispensable part in the rise of human civilization, evolution of modern plants and has cornerstone importance for the sustained long term health and stability of managed and unmanaged ecosystems. Despite its importance, the molecular mechanisms involved in wood formation are still poorly understood.

Wood formation initiates in a lateral meristem known as vascular cambium which consists of meristem cells (cambial initials) organized in single-cell radial file that form continuous cylinder around the stem. Cambium initials divide to produce xylem, phloem, and ray mother cells that in turn undergo several rounds of divisions followed by differentiation into respective cell types. Because of their importance to regulation the quantity and quality of wood production these processes are of substantial theoretical and applied interest but are poorly understood and thus very difficult to manipulate. The progression from meristem to fully differentiated cells is a complex process requiring highly organized transition from different developmental states, most prominently, loosing the pluripotency of meristem cell and committing to a specific cell/tissue fate (e.g., xylem, phloem or rays). Because of the importance of this transition plants establish developmental boundaries that insulate cells in different stages of this progression. We have recently shown that plant-specific transcription factors of the LATERAL ORGAN BOUNDARIES DOMAIN (LBD) family play key role in the establishment and function of these boundaries [The Plant Cell, 2010, 22: 3662–3677].

The discovery leading to the realization of the importance of LBD proteins in woody development was the identification and subsequent characterization of an activation tagged mutant that shows enhanced woody growth. We have found that the activation of a LBD encoding gene, *PtaLBD1* (*Populus tremula x P. alba LBD1*) with uncharacterized previously function in poplar and *Arabidopsis*, is primarily involved in differentiation of secondary phloem and ray cells. Transgenic gain and loss-of-function experiments demonstrated that PtLBD1 is a positive regulator of secondary phloem growth and development. Expression and localization experiments showed predominant expression in the phloem and localization of the transcript on the boundary between the differentiating phloem and the cambium zone. Genes encoding regulators of meristem identity genes like *ARBORKNOX1* (*ARK1*) and *ARBORKNOX2* (*ARK2*) were downregulated while key regulators of phloem initiation like *ALTERED PHLOEM DEVELOPMENT* (*APL*) gene was upregulated in the activation tagged mutant line. The phenotype, localization and misexpression of *ARK*1, *ARK2* and *APL* genes indicate that LBD1 is a critical component in the establishment and function of the developmental boundary between the vascular cambium and the differentiating phloem. It shows that the boundary genes like LBD have dual function to repress meristem identity but promote proliferation and differentiation. We have also found that LBD1 is downregulated by auxin and is co-expressed with strong repressors of auxin response. Because auxin concentrations peak in the cambium and LBD1 is downregulated by auxin we hypothesize that part of the molecular mechanism that establishes the boundary is through auxin-mediated repression of LBD1 in the cambium zone.

Vascular cambium of most extant plants is bifacial – producing xylem to the inside and phloem to the outside of the stem trunk. We then asked if the boundary on the xylem side of vascular cambium is organized and regulated in a similar manner. A broad preliminary survey of the LBD family in *Populus* identified 57 members; microarray results display that four LBD genes (*PtaLBD1*, *PtaLBD*4, *PtaLBD15*, *PtaLBD18*) show predominant expression in stems undergoing wood production with two genes expressed in the phloem (*PtaLBD1* and *PtaLBD*4) and two in the xylem (*PtaLBD15*, *PtaLBD18*). This suggests that a similar regulatory mechanism is in operation during xylem growth and differentiation.

Our studies also shed light on possible mechanisms in the evolution of woodiness and wood anatomy. The LBD proteins are present in most genomes of the sequenced species from the plant kingdom with exceptions of algae. This suggests that the LBD gene family has evolved after the colonization of land plants. Comparative sequence analysis of putative LBD1 orthologs in several sequenced plant genomes, indicates a large and highly significant expansion of the LBD1 gene in *Vitis vinifera*. There were 12 putative LBD1 orthologs. *Vitis* is vine and has a unique wood anatomy. Most notably, the rays are highly multiseriate and secondary phloem is well-developed with multiple growth rings. The intercalation of less-lignified tissues in the xylem of vines and lianas and proportionally more parenchyma tissues is an adaptive feature allowing more stem flexibility. The multiseriate rays and increased secondary phloem production in *Vitis* resembles the poplar transgenics with increased *PtaLBD1* expression. Thus the putative increased LBD1 gene dosage in *Vitis*, well-corresponds to its wood anatomical features and suggests that LBD genes like LBD1 may have played important role in evolution of wood anatomy as an adaptive mechanism reflecting species biology and life habit.

Our findings have broad importance with respect to regulation and evolution of wood formation. They provide tools for manipulation of woody growth and development and provide clues for the mechanisms involved in evolution of wood anatomy with relation to plants’ biology and ecology.

